# Diagnostic efficacy of novel cephalometric parameters for the assessment of vertical skeletal dysplasia

**DOI:** 10.34172/joddd.2022.028

**Published:** 2022-11-15

**Authors:** Kaveri Kranti Gandhi, Anshu Rai

**Affiliations:** ^1^Department of Periodontology, Indiana University School of Dentistry, Indianapolis, USA; ^2^Department of Orthodontics and Dentofacial Orthopedics, Rama Dental College-Hospital and Research Centre Kanpur, Uttar Pradesh, India

**Keywords:** Cephalometry, Dysplasia, Mandible, Maxilla, Orthodontics

## Abstract

**Background.** An accurate diagnosis of vertical skeletal abnormalities presents several challenges. Specific cephalometric parameters can be effectively used for this purpose; however, the diagnostic accuracy of these parameters has not been entirely ascertained. This study examines the effectiveness of two novel cephalometric parameters for diagnosing vertical dysplasia.

**Methods.** In this retrospective study, orthodontic patients were distributed into three study groups: average growth (AGG), horizontal growth (HGG), and vertical growth (VGG). The efficacies of the sum of angles (maxillary, mandibular, and ramal) and the height ratio (lower anterior facial height [LAFH]/upper anterior facial height [UAFH]) in identifying different growth patterns were examined. Receiver operating characteristic (ROC) curves were employed to assess the diagnostic precision quantitatively.

**Results.** A total of 150 patients were included and divided equally among the three study groups. The ramal and mandibular angles varied across AGG, HGG, and VGG; however, the maxillary angle and the sum of these three angles did not vary significantly. There was a substantial variance in LAHF, UAHF, and their ratio in the three groups. The height ratio had 88% and 92% sensitivity to diagnose VGG and HGG, with cut-off values of 46 and 34, respectively (*P*<0.001).

**Conclusion.** Height ratio values varied considerably depending on the facial growth patterns, suggesting its efficacy as a diagnostic tool for skeletal dysplasia, with greater reliability for positive treatment outcomes.

## Introduction

 Vertical growth (VG) disorders present several diagnostic and treatment challenges for orthodontists. Consequently, treating sagittal inconsistencies and, to a lesser degree, transverse disparities has aroused considerable research interest.^1,2^ However, the literature is relatively scant concerning the diagnosis and management of vertical abnormalities.^3,4^ We attempted to investigate novel indices for diagnosing skeletal patterns in the vertical direction to address these limitations. In this study, the validity of two cephalometric indices, the sum of angles, and the ratio of dental heights, in the diagnostic assessment of vertical development was investigated in a group of orthodontic patients from northern India. The receiver operating characteristic (ROC) features of these two indices were also thoroughly investigated.

## Methods

 Patients who underwent orthodontic treatment at the Department of Orthodontics and Dentofacial Orthopedics between 2011 and 2016 were included in this study. The sample size was estimated at 100 using G*Power 3.1.9.4 software, and 150 individuals were recruited to compensate for any dropouts during the study. The inclusion criteria for the patients were those between the ages of 16 and 25, with no known cleft or syndromic conditions and without prior orthodontic treatment. Patients with severe skeletal malocclusion and prior orthognathic surgery or trauma were excluded. To ensure randomization, computer-generated random numbers were used. Each patient was informed about the procedure, and informed consent was obtained to participate in the investigation. The study was carried out according to the ethical principles in the Declaration of Helsinki.

###  Cephalometric evaluation

 A single examiner traced and annotated the landmarks on the patient’s pretreatment lateral cephalograms as a diagnostic tool for treatment planning. Cephalograms were traced and then classified into average (normal), horizontal, and vertical growers using parameters, i.e., the Y-axis, SNGoGn, and the Jarabak ratio.^5,6^

###  Definitions 

**Figure 1:**
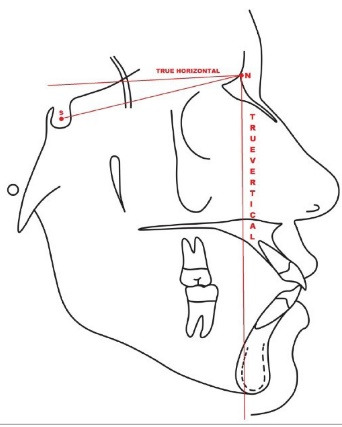
Diagrammatic representation of the true vertical plane (S: sella, N. nasion)

**Figure 2:**
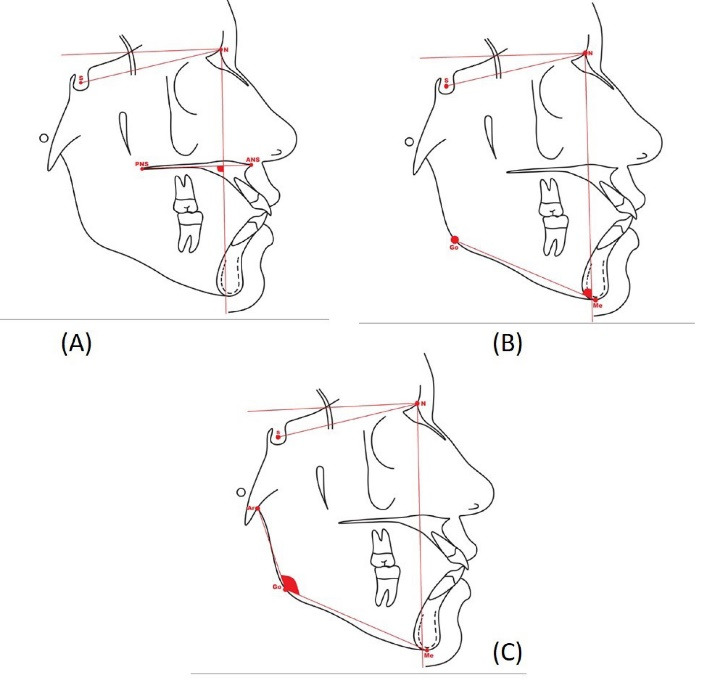
Diagrammatic representation of (A) maxillary angle, (B) mandibular angle, (C) ramal angle (ANS: anterior nasal spine; PNS: posterior nasal spine; N: nasion; S: sella; Go: gonion; Me: menton)

 The Y-axis represents the intersection of the sella-gnathion with the Frankfort horizontal plane. SNGoGn represents the angle between the sella-nasion and the mandibular plane. The angle of SNGoGn defines the behavior of the mandible relative to the cranial base. Jarabak’s ratio is the ratio of the posterior (sella-gonion) and anterior facial heights (nasion-menton). Typically, a ratio of<62% expresses a VG pattern, while>65% expresses horizontal growth (HG). The true vertical plane is the vertical plane formed perpendicular to the nasion by drawing the true horizontal 7 degrees to the sella-nasion plane. The true vertical plane extends to the chin and can be used to measure the maxillary, mandibular, and ramal landmarks relative to it (Figure 1). The maxillary angle is formed between the line constructed by joining posterior nasal spine (PNS) to anterior nasal spine (ANS) and the true vertical plane. This angle represents the maxilla relative to the true vertical plane (Figure 2A). The mandibular angle is the angle between the mandibular plane formed by joining the gonion-menton plane and the true vertical plane. This angle depicts the rotation of the mandibular body relative to the true vertical plane (Figure 2B). The ramal angle is determined by measuring the relation of articulare-gonion-menton. It provides the high- or low-angle relation of the mandible (Figure 2C).

**Figure 3:**
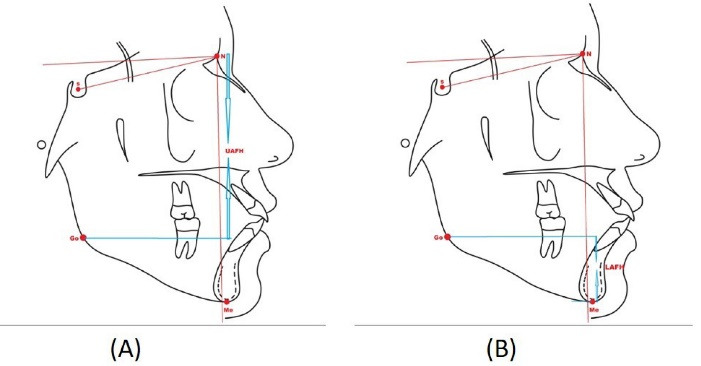
(A) upper anterior facial height (UAFH), (B) lower anterior facial height (LAFH) (N: nasion; S: sella; Go: gonion; Me: menton)

 The upper anterior facial height (UAFH) is a direct measurement taken from the nasion to the gonion along the true vertical plane (Figure 3A). The lower anterior facial height (LAFH) is a direct measurement taken from gonion to menton along the true vertical plane (Figure 3B).

###  Calculations

 Multiple cephalometric parameters are generally used to determine abnormal growth patterns. For example, the mean of the cranial flexure angle (N-S-Ar), the articular angle (S-Ar-Go), and the gonial angle (Ar-Go-Me) can be correlated with VG and HG patterns. In this work, we used the sum of the maxillary, mandibular, and ramal angles to develop an effective index for diagnosing VG (Figure 2A-C).


(i) Sum of angles=Maxillary angle+Mandibular angle+Ramal angle

Another index used in this work to measure the VG pattern was the lower and anterior facial heights ratio (Figure 2A-B). It was expressed as a percentage.


(ii) $$Height\;ratio\left(\%\right)=\frac{LAFH}{UAFH}\times 100$$

###  Statistical analysis 

 Continuous measurements were reported in mean ± SD (Min-Max), and categorical measurements in numbers (%). Significance was measured at a 5% level. The average growth group (AGG), the horizontal growth group (HGG), and the vertical growth group (VGG) were compared to establish a range of values for each sample group to obtain a new parameter to identify vertical skeletal dysplasia. Student’s *t* test (two-tailed, independent) was employed to gauge the significance of study variables on a continuous scale between two groups (intergroup analysis) in the metric parameters. To determine the homogeneity of variance, Levene’s test was used. The chi-squared test was used to determine the significance of categorical parameters. Finally, ROC analysis was used to examine the diagnostic efficacy of these parameters.

## Results

 The age of the participants was 18.19 ± 4.14 years, with 68.7% falling between 11 and 20 years. The remaining participants (31.3%) were aged 21‒30. Among the 150 participants, 50 were assigned to each of the three growth pattern groups: AGG, HGG, and VGG (Table 1). The age of the participants in different groups did not vary significantly (*P* = 0.215, ANOVA).

**Table 1:** Group-wise age distribution of patients included in the study

**Age in years**	**AGG**	**VGG**	**HGG**	**Total**
11-20, n (%)	34 (68%)	31 (62%)	38 (76%)	103 (68.7%)
21-30, n (%)	16 (32%)	19 (38%)	12 (24%)	47 (31.3%)
Total, n (%)	50 (100%)	50 (100%)	50 (100%)	150 (100%)
Mean ± SD	17.36 ± 4.34	18.52 ± 3.89	18.70 ± 4.14	18.19 ± 4.14

AGG: average growth group; HGG: horizontal growth group; VGG: vertical growth group.

 The mean values of the maxillary, mandibular, and ramal angles in each research group are shown in Table 2. The maxillary angle in all three groups was remarkably comparable (*P* = 0.287). On the other hand, the mandibular angle varied significantly between the study groups. VGG had a mandibular angle of 54.41 ± 5.03º, whereas HGG had much higher values of the mandibular angle of 70.68 ± 4.45º. The AGG had intermediate values. The ramal angle was the highest in the VGG and lowest in the HGG. Interestingly, there were no statistically significant differences between the study groups when considering the sum of angles (*P* = 0.225).

**Table 2:** Comparison of maxillary, mandibular, ramal, and sum of angles

	**AGG**	**VGG**	**HGG**	***P*** ** value**
Maxillary angle (^o^)	90.69 ± 3.46	90.91 ± 3.04	90.00 ± 2.38	0.287
Mandibular angle (^o^)	61.10 ± 2.77	54.41 ± 5.03	70.68 ± 4.45	<0.001*
Ramal angle (^o^)	127.06 ± 4.52	132.54 ± 4.46	119.12 ± 6.30	<0.001*
Sum of angle	278.73 ± 5.54	277.78 ± 5.25	279.62 ± 5.11	0.225

AGG: average growth group; HGG: horizontal growth group; VGG: vertical growth group; *P value is based on Anova Test.

 UAFH was the highest in the HGG group and lowest in VGG (*P*<0.001); conversely, LAFH was the lowest in HGG and highest in VGG, with a statistically significant difference between the groups (*P*<0.001). The height ratio further accentuated the difference. The ratio was 51.31 ± 8.25 in VGG and just 25.80 ± 6.50% in HGG; AGG had a ratio of 41.35 ± 4.80% (*P*<0.001) (Table 3).

**Table 3:** UAFH, LAFH, and height ratio in different groups

	**AGG**	**VGG**	**HGG**	**Total**	***P*** ** value**
UAFH	79.22 ± 5.75	76.36 ± 4.89	86.40 ± 6.34	80.66 ± 7.07	<0.001*
LAFH	32.53 ± 3.23	39.47 ± 6.40	22.21 ± 5.28	31.40 ± 8.76	<0.001*
Height ratio	41.35 ± 4.80	51.31 ± 8.25	25.80 ± 6.50	39.49 ± 12.44	<0.001*

AGG: average growth group; HGG: horizontal growth group; VGG: vertical growth group; LAFH: lower anterior facial height; UAFH: upper anterior facial height; *P value is based on ANOVA test.

 To further examine the diagnostic significance of these variables, the sensitivity and specificity were determined using the ROC curve. As expected, the sum of the angle did not yield statistically significant diagnostic values (AUROC = 0.53, *P* = 0.605). In contrast, the height ratio was found to have significant ROC characteristics. The specificity to predict VGG was 88%, with a sensitivity of 76.0% (AUROC-0.0855; *P*<0.001) (Table 4). The height ratio in horizontal growers with a cut-off value of 34.14 had a sensitivity of 92.0% and a specificity of 98.0% (Table 5), suggesting that the height ratio value between 34% and 46% will fall into the category of average growers.

**Table 4:** ROC curve analysis to predict vertical growth

**Variables**	**ROC results to predict VG**				**Cut-off**	**AUROC**	**SE**	***P*** ** value**
	**Sensitivity**	**Specificity**	**LR**^+^	**LR**^-^				
Height ratio (%)	76.00	88.00	6.33	0.27	>46.05	0.855	0.038	<0.001*
Sum of angles (^o^)	90.00	22.00	1.15	0.45	≤ 284	0.530	0.058	0.605

VG: vertical growth; AUROC: area under the receiver operating characteristic; LR: likelihood ratio; SE: standard error. *P value is based on Anova Test.

**Table 5:** ROC curve analysis to predict horizontal growth

**Variables**	**ROC results to predict HG**				**Cut-off**	**AUROC**	**SE**	***P*** ** value**
	**Sensitivity**	**Specificity**	**LR**^+^	**LR**^-^				
Height ratio (%)	92.00	98.00	46.00	0.08	≤ 34.14	0.986	0.008	<0.001
Sum of angles (^o^)	52.00	66.00	1.53	0.73	>280.00	0.562	0.058	0.289

HG: horizontal growth; AUROC: area under the receiver operating characteristic; LR: likelihood ratio; SE: standard error.

## Discussion

 A combination of abnormalities in the maxilla and mandible, exacerbated by other defects, generally leads to vertical dysplasia. To offer effective treatment for individuals with a hyperdivergent skeletal phenotype, a definitive diagnosis is required^7^; however, there is still much debate about the diagnostic usefulness of the metrics used in VG evaluation. This study investigated the diagnostic efficacy of dentoalveolar heights (UADH and UPDH), as well as of the maxillary angle, mandibular angle, and ramal angle, in assessing vertical skeletal dysplasia. This study involved three groups (AGG, HGG, and VGG) defined using specific criteria: the Y-axis, the SNGoGn, and the Jarabak ratio.^5,6,8^

 Our findings did not show differences in the mean values of the maxillary angle between AGG, HGG, and VGG. This may seem to contradict studies suggesting that the location of maxillary incisors can be used to estimate facial abnormalities.^9,10^ In our study, the maxillary angle was measured relative to the true vertical plane. However, the maxillary incisors’ proclivity has traditionally been assessed using cephalometric analysis of the incisor’s long axis (the line that connects the incisal tip to the apex) and planes such as the palatal, sella-nasion, or Frankfort horizontal. The optimal inclination of the maxillary incisor, according to Naini et al,^11^ is approximately parallel to the actual vertical line. Schudy^12^ suggested that the maxillary and mandibular incisors must be adjusted to obtain the perfect interincisal angle to establish functional harmony. Traditional lateral cephalograms or dental casts are often employed to examine the inclination of the maxillary incisors. Despite these studies, the connection between the position of the maxillary incisors and its impact on the facial growth pattern is not conclusive. Notably, the FA angle (90 ± 3.5°) determined by Ricketts’ research is formed between the nasion-basion and the line ranging from the foramen rotundum to the produced gnathion. The FA angle of a retrusive chin is less than that of a protrusive or forward-growing chin. The angle between FA and NBa does not vary with growth; however, it does show the direction of growth and differs between vertical and horizontal growers. The higher angle of the mandibular plane was thought to be a predictive criterion for identifying craniofacial growth direction. Compared to the mandible with an HG, the VG was reported to manifest decreased ramus height, lesser mandibular deepness, augmented gonial angle, and reduced mandibular arc angle.^13^ The gonial angle is considered a promising tool to diagnose the VG or HG pattern; however, the mandibular plane angle is one of the most commonly used parameters for VG, and it is known to be higher in VG than in HG.

 Various cephalometric and non-cephalometric methods have been reported to assess the vertical pattern of a person; however, research does not define a single reliable parameter to allow an easy diagnosis of the discrepancy in the vertical plane, and different values can be obtained for some of these techniques for the same patient, resulting in difficulty in diagnosis and treatment. Tweed^14^ first established the Frankfort-mandibular plane angle in 1946. Björk and Skieller^15,16^ examined the clinical implications of the interrelations and abnormalities among maxillary, mandibular, and the sella–nasion planes. Individuals with class I and II malocclusion divisions were studied by Ngan et al^17^ to examine differences in skeletal changes. Buschang and Martins^18^ found that the vertical and anterior-posterior connections do not remain consistent throughout the growth phase and differ depending on the age, sex, and type of malocclusion. According to the findings of Chung and Wong,^19^ who examined the skeletal and dental morphology of 85 Class II untreated patients, all the groups experienced a reduction in mandibular plane angle and a counter-clockwise rotation of the mandible; however, those with reduced mandibular plane angle experienced more significant rotation.

 Facial growth anomalies become increasingly noticeable as one ages. It has also been demonstrated that dentoalveolar bone develops and changes with age.^20^ The growth of the mandible and maxilla and the alveolar processes govern the VG. VG anomalies can cause vertical malocclusions that tend to worsen with time. Our goal was to create indices generally applicable to a wide age range; therefore, the participants varied in age from 10 to 30 years. Our study showed a marked change in the mandibular angle between VGG and HGG. Furthermore, the ramal angle differed between VGG and HGG; however, it followed a different trend from the mandibular plane angle, with the values of the ramal angle in VGG being higher than those in HGG. There was insignificant variation in the growth patterns when the cumulative values of the three angles were used. ROC analysis revealed that the sum of these angles did not have discernible diagnostic utility. A few other studies found that a large mandibular plane angle was not a strong predictor of facial maturation.^21,22^ Lambrechts et al^23^ found a substantial difference in different cephalometric indices, suggesting the type of mandibular development in two groups with extreme notch depths. Similar results were reported in a few additional implant investigations.^16,24^ In contrast, Kolodziej et al^25^ found a negative association between mandibular antegonial notch depth and growth of the horizontal jaw. We tried using the sum of angles to avoid these restrictions, but it did not provide any diagnostic information. Taking into account our results and other studies mentioned above, it is necessary to conduct more extensive studies involving different age groups, regions, and ethnicities to ascertain the diagnostic utility of the angles discussed above. It may be noted that when the vertical development of the condyle exceeds the sum of the growth of the vertical component of the facial sutures and the alveolar process, the mandible moves to the front of the skull. A convergence of HG and VG produces the final growth vector.

 Individuals with horizontal development patterns had a lower anterior facial height, while people with vertical development patterns had a lower posterior facial height. In our study, UAFH was significantly higher in HGG, and LAFH was significantly lower. A synergistic improvement was seen when the ratio was used, as shown by almost double values achieved in HGG compared to VGG. When this ratio was used in the ROC analysis, both HGG and VGG showed substantial diagnostic efficacy. It should be mentioned that a strong connection between dentoalveolar height and vertical parameters has been found in the literature.^26^ However, our results suggest that using a ratio instead of a number may result in a higher diagnostic value.

 The ratio of the heights of the posterior face to the anterior face is expected to be 65% in normal facial development. Several studies have looked at the connection between dentoalveolar heights and various face typologies, with contradictory findings.^3,15,27,28^ It was reported that male participants’ SN- angles had a positive relationship with either their maxilla height or their mandibular molar area; however, female participants’ angles have no significant relationship with any of these measurements.^29^ Individuals with a large angle (SN-), on the other hand, had low posterior dentoalveolar heights, according to Betzenberger et al.^1^ The reported variations in the studies might be attributed to variances in race, ethnicity, geography, and inclusion criteria.

## Conclusions

 In orthodontic patients from north India, there was a marked variation in certain cephalometric characteristics between individuals with HG, VG, and normal development patterns. Although the ramal and mandibular angles differed substantially in the vertical, horizontal, and normal growth patterns, the maxillary angle and the total of these three angles did not. The sum of angles did not have a significant diagnostic value. In particular, LAHF, UAHF, and their ratio differed significantly between patients with horizontal, vertical, and normal growth patterns. In fact, the height ratio was almost 90% sensitive to identifying horizontal and vertical development patterns. The ratio demonstrated significance, with 34% considered normal,<34% considered horizontal, and>46% considered vertical. Importantly, the height ratio can be calculated using a few cephalometric landmarks that can be easily and precisely identified on digital lateral cephalograms. More research is required to establish the relationship between these cephalometric characteristics and vertical facial growth in individuals with various skeletal malocclusions.

 Our study offers compelling evidence for using the anterior height ratio as a diagnostic tool for VG and HG assessment, which has direct use in clinical practice. This ratio can allow physicians to assess the vertical skeletal disparity quickly, helping with proper diagnosis and treatment planning. Orthodontists may use this information to change the position of the appropriate tooth to minimize the skeletal mismatch responsible for VG, eliminating the need for excessive compensatory motions. Hyperdivergent individuals with open bites may benefit from upper molar intrusion and lower incisor extrusion as treatment. The posterior dentoalveolar heights of the maxilla and mandible may be adjusted to bring the LAFH back within the normal range.

## Acknowledgments

 The authors thank the editors of www.editverse.com for their help with manuscript proofreading and language editing.

## Availability of Data and Materials

 The data analyzed during this study are available from the corresponding author upon reasonable request.

## Author Contributions

 Conceptualization: KKG; Data analysis and interpretation: KKG and AR; Writing: KKG; Review: AR.

## Ethics Approval

 The study protocol was approved by the institutional ethical committee of Rama Dental College, Uttar Pradesh, India. The study was conducted in accordance with the ethical standards specified in the Helsinki Declaration.

## Competing Interests

 None to disclose.
